# A surfactant polymer dressing potentiates antimicrobial efficacy in biofilm disruption

**DOI:** 10.1038/s41598-018-19175-7

**Published:** 2018-01-17

**Authors:** Piya Das Ghatak, Shomita S. Mathew-Steiner, Priyanka Pandey, Sashwati Roy, Chandan K. Sen

**Affiliations:** 1 Department of Surgery, Davis Heart and Lung Research Institute, Center for Regenerative Medicine & Cell-Based Therapies, Wexner Medical Center, The Ohio State University, Columbus, 43210 United States of America; 2 Center for Regenerative Medicine and Cell-Based Therapies, The Ohio State University, Columbus, 43210 United States of America

## Abstract

A 100% water-soluble surfactant polymer dressing (SPD) that is bio-compatible and non-ionic has been reported to improve wound closure in preliminary clinical studies. The mechanism of action of SPD in wound healing remains unclear. Biofilm infection is a significant problem that hinders proper wound closure. The objective of this study was to characterize the mechanism of action of SPD inhibition of bacterial biofilm development. Static biofilms (48 h) of the primary wound pathogens *Pseudomonas aeruginosa* (PA01), *Staphylococcus aureus* (USA300) were grown on polycarbonate membranes and treated with SPD with and without antibiotics for an additional 24 h. The standard antibiotics – tobramycin (10 μg/ml) for PA01 and rifampicin (10 μg/ml) for USA300, were used in these studies. Following 24 h treatment with and without antibiotics, the biofilms were characterized using scanning electron microscopy (SEM) structural imaging, *in vitro* imaging system (IVIS) proliferation imaging, colony forming units (CFU), viability assay, quantitative PCR (qPCR) for virulence gene expression. Because SPD is a surfactant based dressing, it potentially has a direct effect on Gram negative bacteria such as *Pseudomonas* primarily due to the lipid-based outer membrane of the bacteria. SPD is a surfactant based dressing that has potent anti-biofilm properties directly or in synergy with antibiotics.

## Introduction

Chronic wounds represent a significant burden to patients, health care professionals, and the US health care system, affecting 5.7 million patients and costing an estimated 25 billion dollars annually^1,2^. Bioburden, particularly in the form of microbial ‘biofilms’, is a significant barrier to healing of chronic wounds^3^. By definition, a biofilm is an *aggregate* of microorganisms that are found to be associated with biotic or abiotic surfaces^4^. The aggregate is held together by polymeric matrix secreted by the bacteria themselves^5^. The self-produced matrix helps bacteria to adhere to each other and/or to the substrate surface and serves as a defensive barrier against the penetration of antimicrobial substances and antibodies^6–11^.

Wound debridement has been widely used to remove necrotic tissue from a wound to remove dead and infected tissue and promote healing^12–15^. Necrotic tissue prolongs the inflammatory stage and may serve as a reservoir for biofilm bacteria. Wound debridement may be performed in several different ways: surgical, autolytic, enzymatic, and mechanical^15–18^. Each of these has its own benefits and shortcomings, depending on the wound type and underlying patient health. Furthermore, wound cleansers are often used before or even alongside debridement agents to remove loosened tissue debris, bacteria, and other physicochemical contaminants that can seriously impede the wound healing process. Some dressings contain certain levels of metal elements (*e.g*., silver) as principal bactericides, while wound cleansers rely on the cleaning power of various surfactants to remove the debris from the wound bed. The use of surfactants for biofilm removal as part of chemical treatment has been proposed^19^. Most surfactants are primarily employed as wound scrubs and cleansing solutions^20–26^ and also as carriers for antibiotics and antimicrobials^27^. Poloxamer-based non-ionic surfactant gels have been used as delivery agents for antimicrobials. Compared to the standard silver sulfadiazine creams, surfactant gels are easily removed from wound surfaces^28,29^. Interestingly, poloxamer surfactants have been well tolerated by patients when topically applied^20,21,30,31^. It is claimed that poloxamer may have *pro-healing* effects on full-thickness skin wounds^32^.

The purpose of this current work was to evaluate the effect of a surfactant polymer dressing (SPD) on two primary wound pathogens - *Pseudomonas aeruginosa* PA01 and *Staphylococcus aureus* USA300. USA 300 is a methicillin resistant isolate. SPD is a burn and wound dressing that is 100% water-soluble, poloxamer-based and non-ionic. SPD is generally recognized as safe by the Food and Drug Administration and is used in clinic as clinic as a product that softens, loosens and traps debris and necrotic tissue. In addition to addressing the effect of SPD on PA01 and USA300 in their planktonic forms, this work investigates the potential effects of SPD on biofilm infection and related mechanisms.

## Results

### SPD exhibits anti-bacterial properties

SPD significantly decreased the growth rate of both Gram negative (*P. aeruginosa* PA01) and Gram positive (*S. aureus* USA300) bacteria grown planktonically in broth cultures. Optical density (OD600) measurements indicated slower growth kinetics in SPD treated compared to untreated broth cultures (Supplemental Fig. S1A,B). Viability analysis using CFU/ml calculations indicated significant decrease in SPD treated (10^6^–10^8^) compared to untreated (>10^10^) Gram positive and Gram negative bacterial strains. However, CFU/ml viability assay performed on cultures following 24 h of treatment suggested a bacteriostatic rather than bactericidal effect of SPD. Although viability was significantly decreased in SPD treated samples, the bacteria were still able to grow once the inhibitory effect of SPD was withdrawn (Supplemental Fig. S1C,D).

### Rhl-regulated virulence factor, pyocyanin, inhibited by SPD

**Figure 1:**
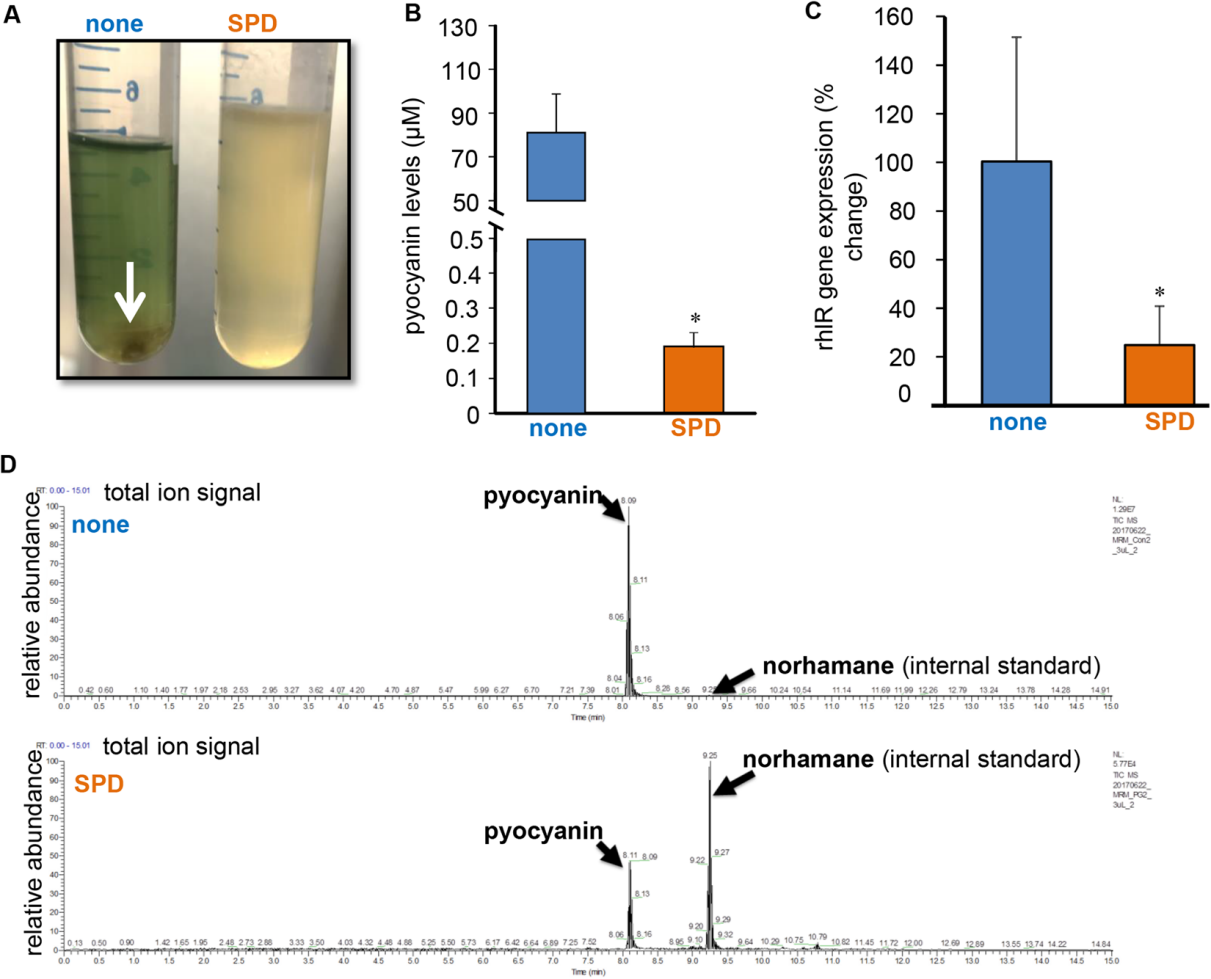
SPD inhibits Rhl regulated pyocyanin production by *P. aeruginosa* PA01. (**A**) Biofilm co-aggregation observed in the no treatment PA01 culture was not observed in SPD treated 48-72 hours cultures, n = 6. (**B**) Bar graph showing mean levels of pyocyanin in control and SPD treated samples. Data are shown mean ± SD, n = 6, *p < 0.05. (**C**) The total ion signal chromatograms of pyocyanin and internal standard norharmane produced by *P. aeruginosa* PA01 in normal condition (con) and in the presence of compound (SPD). The retention times for pyocyanin and norharmane with the solvent system used in this study were 8.1 min and 9.25 min, respectively. The total ion signal chromatograms of pyocyanin and norharmane (internal standard) produced by *P. aeruginosa* PA01 in normal condition (con) and in the presence of compound (SPD). The retention times for pyocyanin and norharmane with the solvent system used in this study were 8.1 min and 9.25 min, respectively. (**D**) *P aeruginosa* PA01 was either left untreated or treated for 24 h with SPD. mRNA was isolated and real-time PCR was performed to assess *rhlR* gene expression in PA01.

During growth curve studies it was observed that PA01 grown in the presence of SPD did not produce the characteristic green pigment pyocyanin after 48–72 h of treatment (Fig. 1A). Pyocyanin is a virulence factor produced by *P. aeruginosa* and is regulated by the *rhl* quorum sensing pathway. Liquid chromatography – mass spectrometry (LC-MS) analysis provided quantitative corroboration of low pyocyanin production in SPD treated samples (Fig. 1C). Furthermore, markedly lowered expression of *rhlR* was observed in SPD treated samples. 16 s rRNA was used as the housekeeping gene. Interestingly, untreated samples also showed characteristic aggregates of bacteria (Fig. 1A, white arrow) that were conspicuously absent in SPD treated cultures. The uniform turbidity of SPD treated cultures point towards the ability of SPD to inhibit aggregation of biofilm forming PA01.

### SPD disrupted biofilm matrix

**Figure 2:**
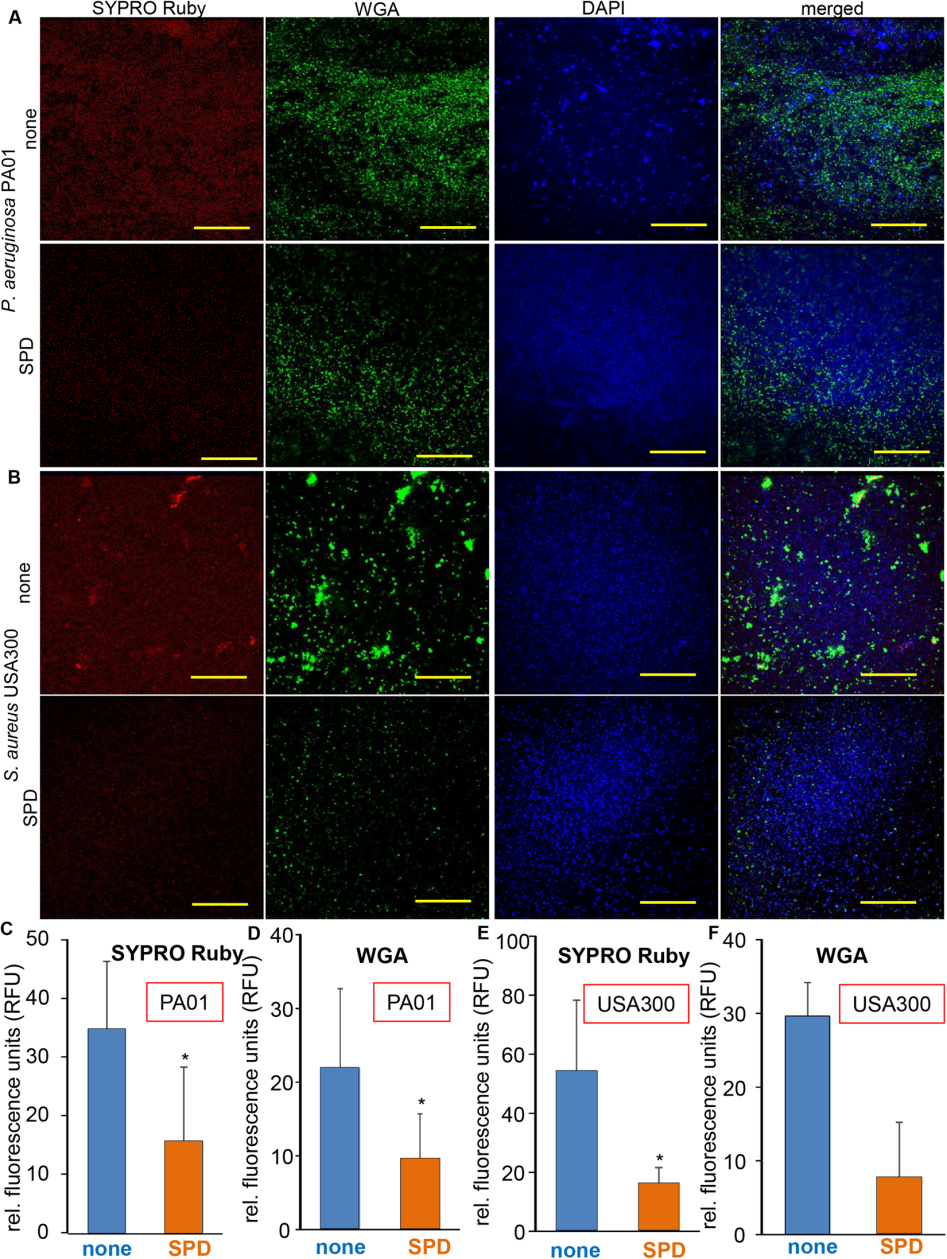
SPD disrupts biofilm matrix in *P. aeruginosa* PA01 and S*. aureus* USA300. Triple staining with DAPI (**DNA**), SYPRO Ruby (**biofilm matrices**) and FITC-Wheat Germ Agglutinin (**membrane glycoproteins**) was performed for *P. aeruginosa* PA01. (**A**) Shown are representative images of PA01 biofilms that were untreated or SPD treated and stained with the respective dyes. Graphical representation of staining intensity for (**C**) SYPRO Ruby and (**D**) WGA are shown. Data are shown mean ± SD, n = 6. *p≤0.05. Scale bar = 40 µM. Triple staining with DAPI (**DNA**), SYPRO Ruby (**biofilm matrices**) and FITC-Wheat Germ Agglutinin (**membrane glycoproteins**) was performed for *S. aureus* USA300. (**B**) Shown are representative images of USA300 biofilms that were untreated or SPD treated and stained with the respective dyes. Graphical representation of staining intensity for (**E**) SYPRO Ruby and (**F**) WGA are shown. Data are shown mean ± SD, n = 6. *p≤0.05. Scale bar = 40 µM.

The observation that SPD inhibited PA01 aggregation in broth culture prompted our studies testing the effect of SPD on biofilm formation. We utilized the static biofilm model as previously published^33^. Bacteria in a biofilm matrix are encased within extrapolymeric substance (EPS) that is typically composed of nucleic acid, proteins and polysaccharides. Confocal microscopic analysis using matrix targeted stains such as SYPRO Ruby (protein components) and wheat germ agglutinin (WGA; cell wall components) indicated significant disruption of PA01 and USA300 biofilms by SPD (Fig. 2). This was visualized qualitatively as minimized positive staining in SYPRO Ruby (red), WGA (green) and DAPI (blue) channels in SPD treated compared to no treatment groups. Quantifications of SYPRO Ruby and WGA staining in PA01 and USA300 biofilms that were untreated or SPD treated indicated significant decrease in matrix components.

### SPD inhibited metabolic activity of biofilm bacteria

**Figure 3:**
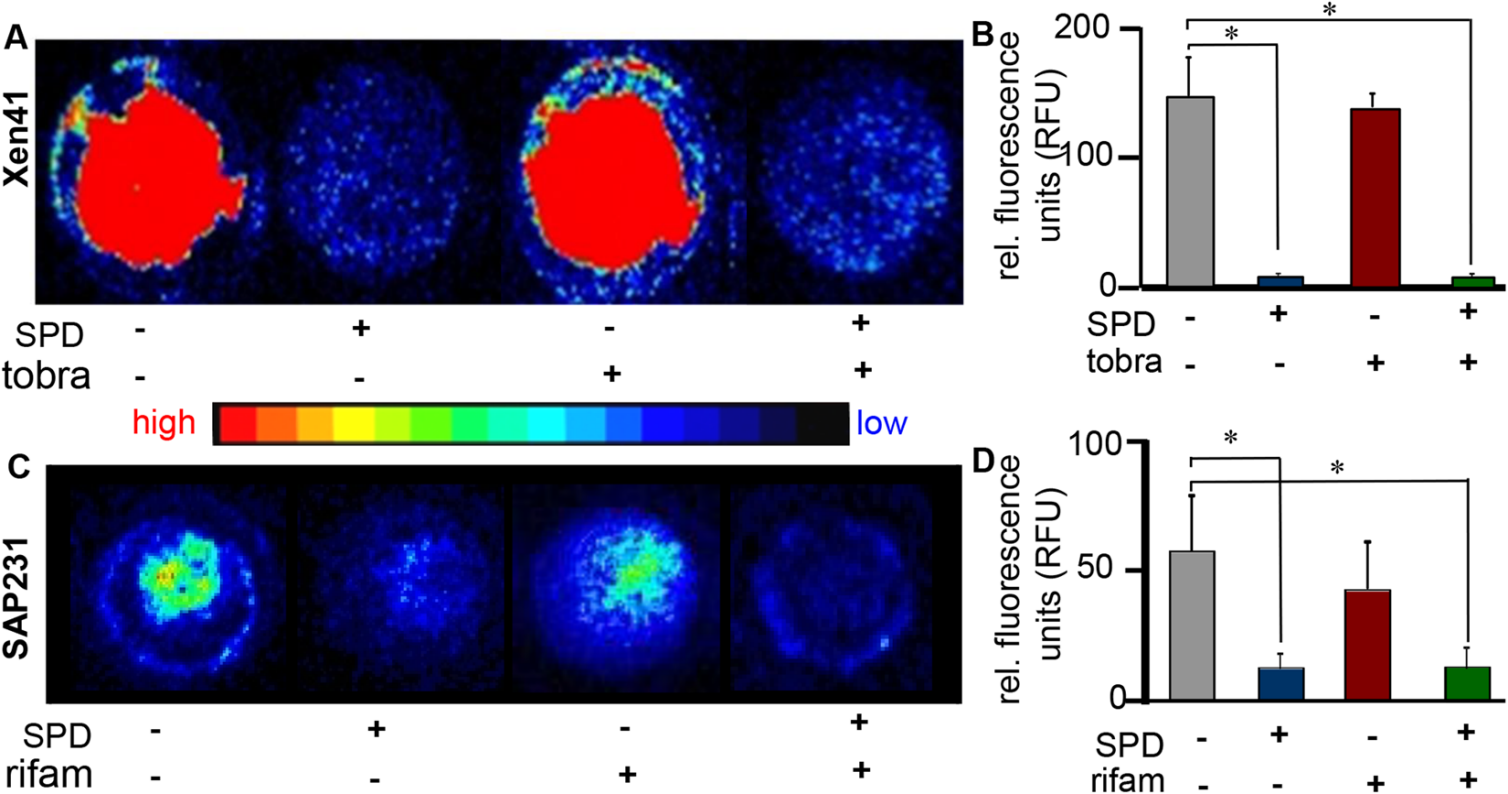
SPD decreases metabolic activity of bacteria. Shown are representative IVIS images and corresponding graphical representations of intensity quantitation from 48 h biofilms of (**A,B**) *P. aeruginosa* Xen41 and (**C,D**) *S. aureus* SAP231 either left untreated, or treated for 24 h with antibiotic (tobramycin (tobra) or rifampicin (rifam) respectively) alone, SPD alone or a combination of SPD and antibiotic. Data are shown mean ± SD, n = 8, *p < 0.05. Also included is a heat map showing intensity ranges. The intensity of blue and red signals were quantified using ImageJ and shows that bacteria in both control and antibiotic treated group show higher metabolic activity whereas SPD and combination therapy resulted in decreased metabolic activity.

The effects of SPD on the metabolic activity of biofilm bacteria were investigated using bioluminescent strains of PA01 (Xen41) and USA300 (SAP231). Static biofilms of Xen41 and SAP231 were treated with SPD or relevant antibiotics alone or together for 24 h. Following 24 h treatment, IVIS imaging indicated that for both strains, SPD alone or in combination with antibiotic caused a significant decrease in bacterial metabolism within the biofilm (Fig. 3A,B) compared to no treatment. Biofilms treated with antibiotic alone (tobramycin for PA01 and rifampicin for USA300) showed metabolic activity similar to that of the control group. To test inhibition of metabolic activity a biochemical index, ADP/ATP ratio, was measured in PA01 (Supplemental Fig. S2). Elevated ADP/ATP in response to SPD indicated metabolic inhibition.

### Synergistic function of SPD and antibiotics against biofilm

**Figure 4:**
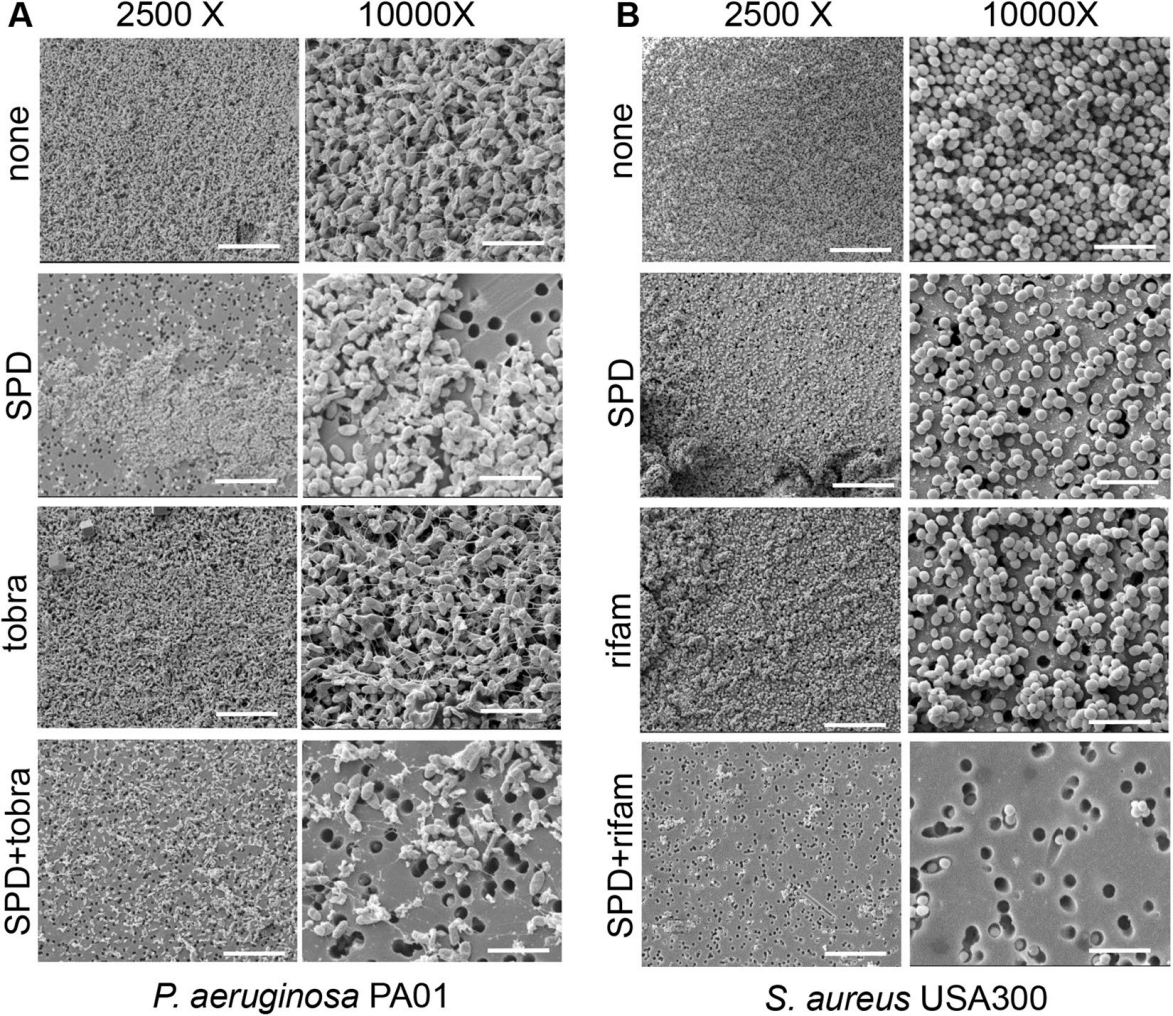
SPD acts synergistically with antibiotics and disrupts biofilm structural integrity. 48 h static biofilms of (**A**) *P. aeruginosa* PA01 and (**B**) *S. aureus* USA300 were either left untreated or treated for 24 h with SPD or antibiotic (tobramycin (tobra) or rifampicin (rifam)) alone or in combination. Shown are representative scanning electron micrographs (SEM) at 2500× and 10000× magnifications. SPD alone or in combination with antibiotics showed fewer dense aggregates of bacteria with associated matrix. n = 6. Scale bar = 10 μm, 2500× magnification and 5 μm, 10000× magnification.

When SPD was applied to static biofilm of PA01 and USA300, impaired cell viability was observed. Such antimicrobial effect was further augmented in the presence of appropriate antibiotics (Fig. 4 and Supplemental Fig. S3). Compared to no-treatment and antibiotic alone, SPD treated PA01 (Supplemental Fig. S3A) and USA300 (Supplemental Fig. S3C) biofilms showed loss of bacteria viability as visualized by confocal microscopy. Such effect was further augmented in the SPD and antibiotic combination treatment group, indicating synergistic action between SPD and antibiotics (Supplemental Fig. S3B, D).

Static (48 h) biofilms of (**A**) *P. aeruginosa* PA01 and (**B**) *S. aureus* USA300 were either left untreated or treated for 24 h with SPD or antibiotic (tobramycin (tobra) or rifampicin (rifam) alone or in combination. Shown are representative scanning electron micrographs (SEM) at 2500× and 10000× magnifications. SPD alone or in combination with antibiotics showed fewer dense aggregates of bacteria with associated matrix. n = 6. Scale bar = 10 μm, 2500× magnification and 5 μm, 10000× magnification (Fig. 2).

## Discussion

Anti-biofilm property of a clinically used wound debridement agent is of major significance in wound care. Surfactants are known to interact with cellular components such as proteins and lipids, resulting in inhibitory effects on the growth and viability of microbial cells^34,35^. Surfactants are also known to induce cell autolysis in bacterial strains such as *Bacillus subtilis*^36–38^ and inhibit respiration in *P. aeruginosa*^39^. Antibacterial agents can elicit both bacteriostatic and/or bactericidal effects. Bacteriostatic effects are reversible following the removal or neutralization of the antibacterial agent^40^. In contrast, bactericidal action results in irreversible or irreparable damage to a vital cell structure or function^41^. Earlier works have reported several anionic and nonionic detergents to have bacteriostatic, but not bactericidal activity^42–47^. In keeping with this, SPD, a surfactant, was found to exert a bacteriostatic effect on the bacterial strains studied. However, in the case of SPD the possibility of bacterial cell death may not be ruled out. Quorum-sensing (QS) is a social behavior exhibited by bacterial strains whereby chemical-based communication modulates behavior of the group/aggregates of bacteria. The production of several virulence factors by *P. aeruginosa* is controlled *via* threshold aggregation through two QS systems, *las* and *rhl*^48^. The *rhl* QS system has been shown to control the expression of the genes coding for pyocyanin, a toxic secondary metabolite, among other important virulence factors. PA01 grown in presence of SPD showed blunted pyocyanin production. It is widely accepted that the ability to communicate both within and between species is critical for bacterial survival and possibly pathogenicity^49–51^. The impact of quorum sensing on virulence, makes it an attractive target for the development of new therapeutic strategies^52^. Our observations are the first to describe a direct effect of SPD on QS. These findings could be attributed to a direct negative effect of SPD on bacterial cell viability and/or aggregation capacity. External application of surfactant disrupted the integrity of biological lipid membranes causing loss of bacterial cell viability. Furthermore, SPD mediated disruption of inter-bacterial physical interactions, could inhibit aggregation and formation of the bacterial quorum needed to activate QS pathways. Detergents are known to be capable of neutralizing adhesion of bacterial aggregates, a key process in biofilm formation^53,54^. Studies testing the effect of SPD on the expression of other *rhl* regulated virulence factors as well as other QS pathways such as *las* and PQS are warranted.

Findings of this work are consistent with previously reported observations claiming anti-biofilm effects of SPD in a porcine skin explant infected with *P. aeruginosa*^55^. A regimen of daily application of SPD resulted in elimination of *P. aeruginosa* biofilms within 3 days through ‘soft’ debridement *via* simple wiping off of the dressing. The use of SPD converted biofilm to a more susceptible planktonic-like phenotype. The ability of detergents to disperse biofilm has been previously reported^56,57^. Findings of this work demonstrating SPD-induced dissolution of biofilm matrix and dismantling of biofilm structure provide a mechanistic basis for the observation made in the pig skin explants study^55^. Bacteria that grow in biofilms have decreased antibiotic susceptibility that further promotes persistent infection in wounds or implanted devices^58,59^. Among the mechanisms responsible for such decreased susceptibility are retarded antibiotic penetration, altered microenvironment and slow growth and activation of protective stress responses^59^. Dispersion of biofilm structures, as evident in this work as well, has been recognized as an important factor that increases antibiotic susceptibility of detergent-treated biofilm bacteria^60–62^. The synergistic effect of combination therapies are rapidly becoming the new alternative strategy to increasing killing potency of antimicrobial agents and controlling bacterial infections^63–65^. Our observations are the first to describe a synergistic inhibition of biofilm viability by SPD with antibiotics. These findings could reflect improved antibiotic penetration and accessibility to bacterial targets within biofilm caused by SPD-mediated disruption of biofilm matrix structure.

In conclusion, SPD is a surfactant based wound dressing that possesses anti-biofilm properties directly or in synergy with antibiotics. Our studies provide initial insight into a possible role for SPD as an anti-biofilm agent. Taken together with its biocompatible and non-toxic nature, SPD may have promising applications in wound care. *In vitro* biofilm studies are useful tools to dissect mechanism of action of agents such as surfactant polymers but require supporting studies in relevant pre-clinical models and controlled clinical studies to ensure that these findings are translationally relevant.

## Materials and Methods

### Surfactant polymer dressing (SPD)

A non-ionic surfactant polymer dressing (SPD; PluroGel®, Medline Industries, Inc.) was used^66^. A 200 mg/ml final concentration was used for the experiments involving suspension culture. For non-suspension biofilm studies, a final concentration of 0.89 mg/mm^2^ of SPD was used.

### Bacterial strains and growth conditions

The strains used for this study included *Staphylococcus aureus* (USA300LAC, the dominant clone of the CA-MRSA lineage) and *Pseudomonas aeruginosa* PA01 (wild-type strain; serotype O5)^67,68^. Additionally, for the IVIS experiments performed, bioluminescent strains of *S. aureus* (SAP231; base strain USA300) and *P. aeruginosa* (Xen41; base strain PA01) (PerkinElmer, USA) were used^69^. Both these strains contain an integrated plasmid carrying a luciferase gene that is constitutively expressed. *S. aureus* strains were cultured in tryptic soy broth (TSB) and *P. aeruginosa* strains were cultured in low salt Luria-Bertani LB broth for planktonic culture and the respective agar formats for biofilm studies (Thermo Fisher Scientific). Bioluminescent bacteria only produce light when they are alive and metabolically active. Thus, we used light emission as detected with the *in vivo* imaging system (IVIS) spectrum system (Perkin Elmer), as an indicator of bacterial activity.

### Bacterial growth curves and CFU analyses

PA01 and USA300 were cultured in round bottom tubes in LB or TSB media, respectively, with continuous shaking @300 rpm at 37 °C. Absorbance was obtained using 10 mm optical path length quartz cuvettes in a UV-Vis spectrophotometer by measuring optical density (OD) at 600 nm over different time points. Colony forming units (CFU) are indicators of bacterial growth. Following a 24 h growth period with or without SPD, serial dilutions of the broth culture was performed using sterile 1× -phosphate buffered saline (PBS). A standard volume of select dilutions were plated on agar plates and incubated overnight at 37 °C. Manual colony count was performed and used to calculate total bacterial load per ml of broth (CFU/ml).

### Bacterial growth following SPD withdrawal

To evaluate bacteriostatic properties of SPD, SPD was withdrawn to test whether bacterial growth recovers. PA01 and USA300 were cultured in round bottom tubes in LB or TSB media, respectively, with continuous shaking (300 rpm) at 37 °C for. Following a 10 h growth period with SPD, SPD was withdrawn from the growth media by centrifuging the broth and resuspending the bacterial cell pellet in fresh LB or TSB media. All three groups (broth without SPD, broth exposed to SPD for 10 h and broth treated with SPD for the entire 10 + 24 h duration) were then allowed to grow for an additional 24 h. Absorbance was obtained using 10 mm optical path length quartz cuvettes in a UV-Vis spectrophotometer by measuring optical density (OD) at 600 nm.

### Mass spectrometric analysis of pyocyanin

To prepare *P*. *aeruginosa* conditioned medium, cultures were grown for 3 days to a high optical density (OD), at which quorum-sensing is activated in PA01, thus triggering the production of the cytotoxic exoproduct pyocyanin (PCN)^70–72^. PCN was extracted from cell-free filtrate using methanol (1:1) precipitation. Norharmane (NH) was used as internal standard. After centrifugation the supernatant was diluted 1:10 in 0.1% Formic Acid (v/v) in H_2_O and subjected to HPLC-MS analysis. Liquid chromatography and MS were performed by the Mass Spectrometry and Proteomics Facility (The Ohio State University) on a Dionex 3000 LC unit with a Thermo Quantiva triple quadrupole mass spectrometer equipped with an electrospray ionization source and run in the positive ion mode for detection. Briefly, an aliquot of the broth (fluid) following protein precipitation was added into an Agilent ZORBAX SB-Aq 3.5 µm column (150 × 3.0 mm). The mobile phase gradient was generated using H_2_O with 0.1% formic acid and 100% acetonitrile at a flow of 400 µL/min and a total run time of 15 min. The major PCN peak eluted at 8.1 min while NH eluted at 9.25 min and they were detected from using the following precursor to fragment transitions; for PCN 211.1/168.1, 211.1/183.1, and 211.1/196.1, while NH transitions were monitored at 169.1/115.0 and 169.1/142.1. With a series of standards from 0.001 to 50 µM PCN and an NH internal standard concentration of 0.28 µM the ratio of PCN to NH was determined for each to produce a calibration curve with an R^2^ of 0.9960 and a linear regression of (Area ratio = 1.892*conc in µM).

### Aggregation assay

Multicell auto aggregation of planktonic PA01 cells is a phenotype observed in the initial phase of biofilm formation. Bacteria were grown in LB medium for overnight at 37 °C. Overnight culture was diluted in LB and incubated for 48 h in presence or absence of SPD under same culture conditions. Cells were gently inverted several times and placed at room temperature for 15 min^73^.

### *In vitro* biofilm model

PA01 and USA300 biofilms were developed *in vitro* using a polycarbonate filter model. Grown overnight in LB and TSB media at 37 °C, bacteria were cultured on sterile polycarbonate membrane filters placed on LBA and TSA agar plates, respectively, and allowed to form mature biofilm for 48 h. The biofilms were then exposed to SPD alone, antibiotic (10 µg/ml each of tobramycin or rifampicin) alone and SPD in combination with antibiotic or left untreated for 24 h^73^.

### Scanning electron microscopy (SEM)

Biofilm grown on polycarbonate membranes were fixed in 2% glutaraldehyde solution for 48 h at 4 °C, washed with phosphate-buffered saline (PBS), dehydrated in a graded ethanol series, washed with hexamethyldisilazane (HMDS, Ted Pella Inc., CA) and left to air-dry overnight. The samples were mounted and sputter-coated with a gold-palladium (Au-Pd) and imaged with the SEM operating at 5 kV in the secondary electron mode (XL 30 S; FEG, FEI Co., Hillsboro, OR)^74^.

### Viability staining

The BacLight™ bacterial viability kit for microscopy and quantitative assays were used to monitor bacterial viability. Bacterial cells with a compromised membrane that are considered to be dead or dying stain red, whereas cells with an intact membrane stain green. The polycarbonate membrane discs were visualized using an Olympus Fluoview FV1000 spectral confocal microscope (Olympus FV1000 IX81, ON) under 40× magnification using an argon laser^73^.

### Biofilm matrix staining

Bacterial biofilm were prepared under static conditions as described and stained with FilmTracer™ SYPRO® Ruby biofilm matrix stain (Molecular Probes) according to manufacturer’s instructions^75^. After 30 min incubation at room temperature, the fluorescent marker solution was removed. Biofilm were washed with water. Next, the biofilm discs were stained with wheat germ agglutinin, Alexa Fluor™ 488 conjugate (ThermoFisher Scientific, 5.0 µg/mL) for 10 minutes at room temperature and finally DAPI (Invitrogen 1:10,000) was used as a nuclear counterstain. Stained biofilms were visualized by confocal laser scanning microscopy (CLSM; Olympus FV1000 IX81, ON). Images were analyzed for their relative fluorescence units (RFU) using ImageJ, an application available from NIH at http://imagej.nih.gov/ij/download.html. After loading the image into ImageJ, the channels were split by running Image → Colour → Split and channel intensities were measured^76^.

### Quantification of mRNA expression

Total RNA was isolated using Norgen™ RNA isolation kit, according to the manufacturer’s protocol. Gene expression levels were quantified with real-time PCR system and SYBR Green (Applied Biosystems) and normalized to 16 s rRNA as housekeeping genes. Expression levels were quantified employing the 2 (−ΔΔct) relative quantification methods. The following primer sets were used: *P.aeruginosa* 16 s rRNA (forward) 5′–GGG GGA TCT TCG GAC CTC A–3′, (reverse) 5′–TCC TTA GAG TGC CCA CCC G–3′, pmas_rhlR (forward) 5′–TGC CGT ATC GGC AAG GCT GC–3′, (reverse) 5′–TTC CAG GAC GGC GAA CAC GC–3′^73^.

### ADP/ATP ratio assay

ADP/ATP was measured as marker of metabolic activity. Bacterial cells were collected by vigorously vortexing the polycarbonate membrane discs in assay buffer provided in the ADP/ATP Ratio Assay Kit (BioAssay Systems, Hayward, CA). Luminescence was measured according to the manufacturer’s instructions.

### Statistics

Control and treated samples were compared using a paired *t* test. One-way ANOVA was used for all other comparison of differences between means of multiple samples in a group. *p* < 0.05 was considered significant. Data represented in bar graphs were plotted as mean ± SD. Replicates represent biological replicates.

## Electronic supplementary material

Supplementary figures
