# Supplementary material for: A surfactant polymer dressing potentiates antimicrobial efficacy in biofilm disruption
Source: Sci Rep. 2018 Jan 17;8:873. doi: 10.1038/s41598-018-19175-7 (PMC5772662; doi:10.1038/s41598-018-19175-7)
Supplement: Supplementary file 1 — Supplementary figures [file 41598_2018_19175_MOESM1_ESM.pdf]

Supplementary Information

A surfactant polymer dressing potentiates antimicrobial efficacy in biofilm disruption

Piya Das Ghatak[1,2], Shomita S. Mathew-Steiner[1,2], Priyanka Pandey[1,2], Sashwati Roy[1,2], and Chandan K. Sen[1,2*]

[1]Department of Surgery, Davis Heart and Lung Research Institute, Center for Regenerative Medicine & Cell-Based Therapies, Wexner Medical Center, The Ohio State University, Columbus, 43210, United States of America

[2]Center for Regenerative Medicine and Cell-Based Therapies, The Ohio State University, Columbus, 43210, United States of America

***Corresponding Author:** Chandan K. Sen
Professor & Director
Center for Regenerative Medicine & Cell-Based Therapies
The Ohio State University Wexner Medical Center,
473 West 12th Ave, Columbus, OH 43210.
Tel.: 614-247-7658; Fax: 614-247-7818

E-mail: chandan.sen@osumc.edu

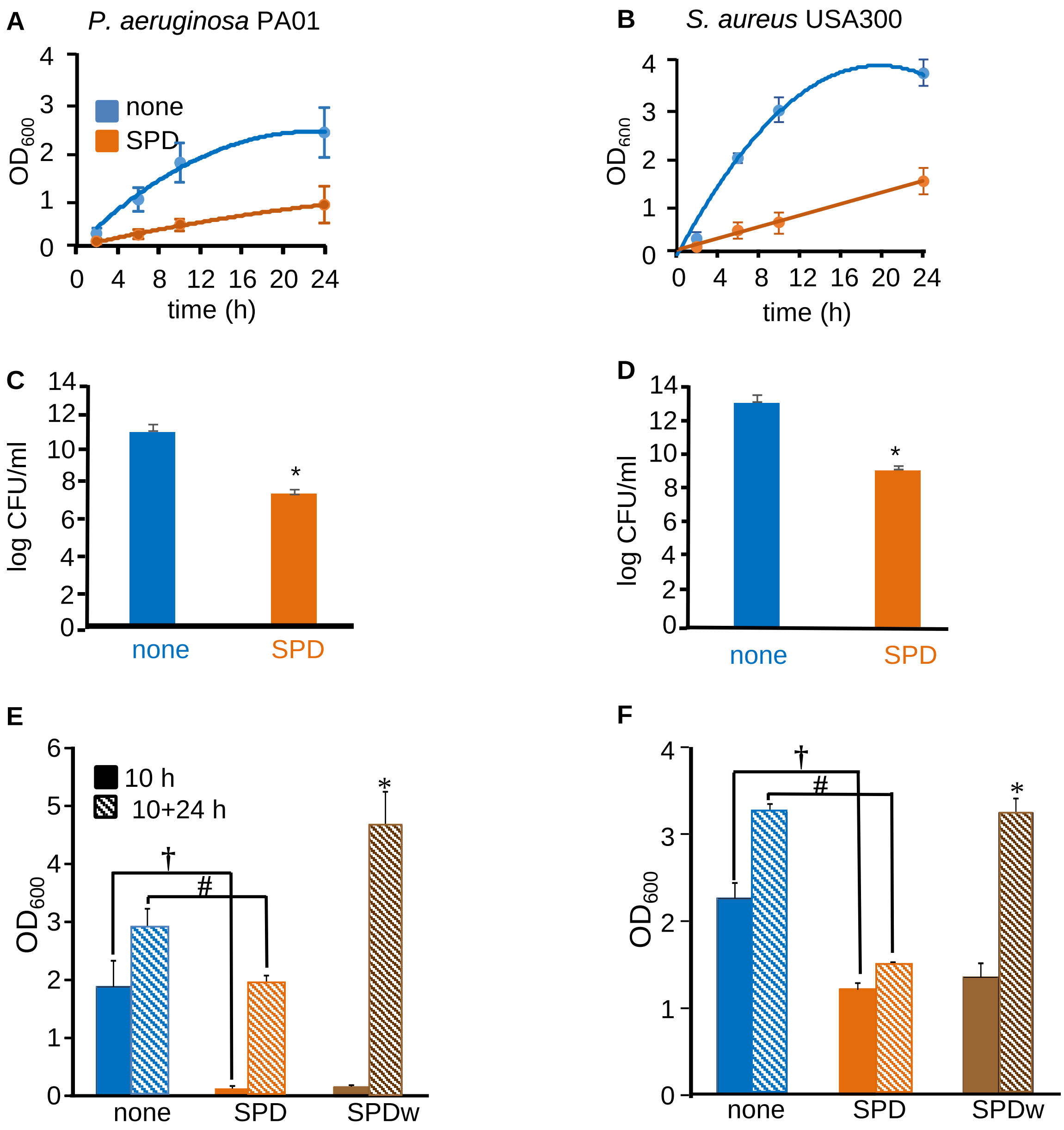

Figure S1. SPD exhibits bacteriostatic properties.
(A - D) Shown are graphical representations of absorbance (OD_{600}) and colony forming unit (CFU/ml) measurements from planktonic *P. aeruginosa* PA01 and *S. aureus* USA300 in presence or absence of SPD. Data are shown mean±SD, n=6, *p<0.05. (E – F) Absorbance (OD_{600}) from planktonic *P. aeruginosa* PA01 and *S. aureus* USA300 in presence or absence of SPD. SPDw indicates withdrawal of SPD after 10h followed by culture for an additional 24h. Data are shown mean ± SD, n=4, *p<0.0005, † p<0.01, #p<0.01.

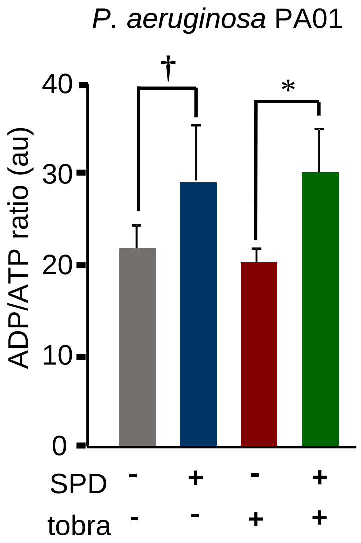

Figure S2. SPD caused metabolic inhibition. Shown are graphical representations of ADP/ATP ratio from *P. aeruginosa* PA01 biofilm in presence or absence of SPD and antibiotic. Data are shown mean±SD, n=6, *p<0.01, † p<0.01. au=arbitrary units

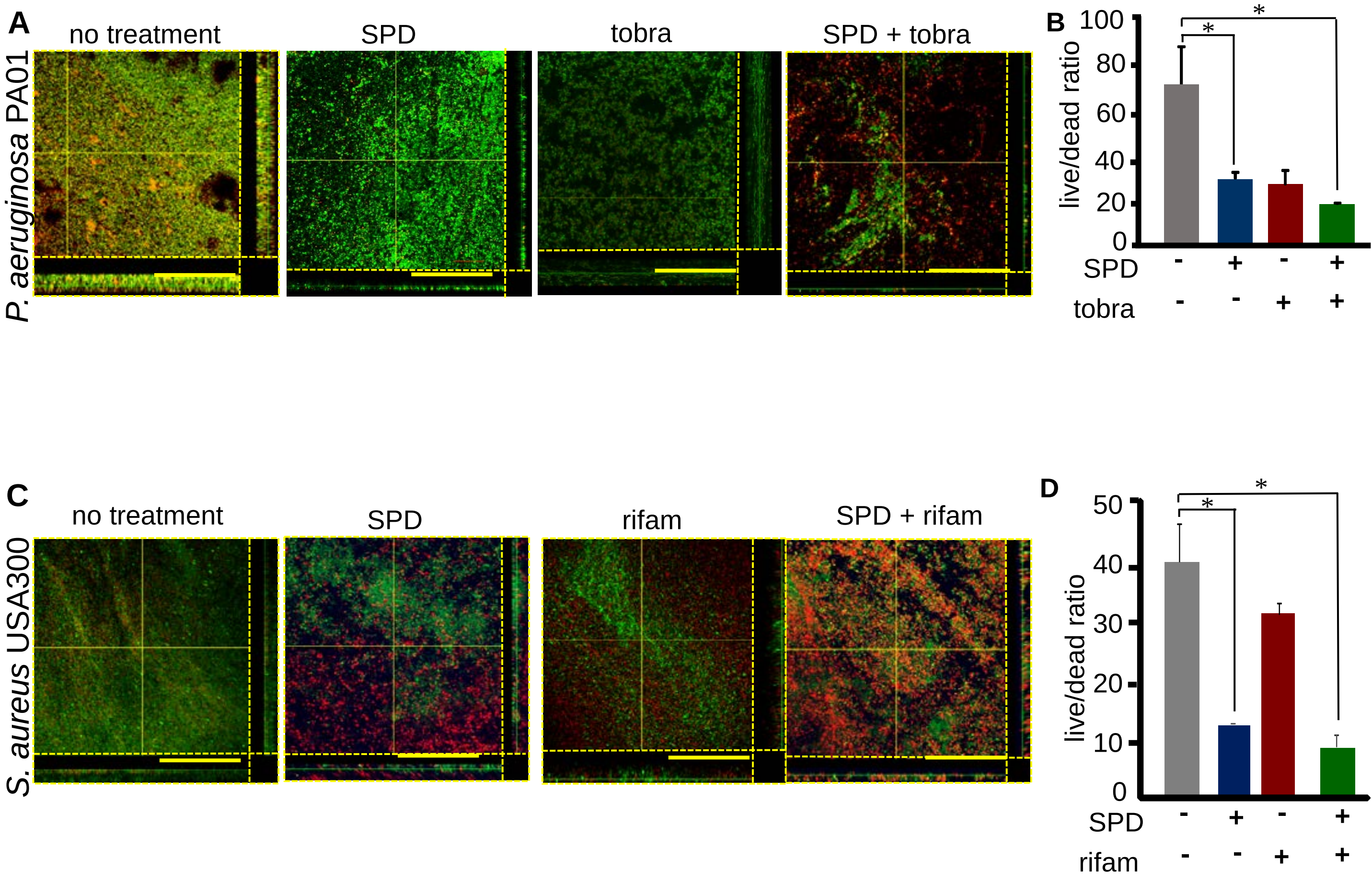

Figure S3. SPD synergizes with antibiotics to impair biofilm cell viability.

(A - D) Fig 6. 48h static biofilms of **(A)** *P aeruginosa* PA01 and **(B)** *S.aureus* USA300 were either left untreated or treated for 24h with SPD or antibiotic (tobramycin (tobra) or rifampicin (rifam) respectively) alone or in combination. Shown are representative CLSM micrographs and corresponding live/dead ratio quantitation. The green fluorescence indicates live bacteria while the red indicates dead bacteria. The intensities of red and green channels were quantified using ImageJ. SPD along with antibiotic showed significant decrease in bacterial viability in both strains tested. Data shown as mean±SD, n = 6, *p<0.05. Scale bar = 40 µM.
